# Analysis of the Supportive Care Needs after Metabolic Bariatric Surgery in Germany Using an Adjusted Supportive Care Needs Survey- Short Form (SCNS-SF34)

**DOI:** 10.1007/s11695-026-08598-1

**Published:** 2026-03-21

**Authors:** Jonas Wagner, Madita Roll, Tobias Kantowski, Sara Notz, Gabriel Plitzko, Jakob Izbicki, Oliver Mann, Thilo Hackert, Anna Duprée, Freya Brodersen, Angelika Weigel

**Affiliations:** 1 Department of General, Visceral and Thoracic Surgery, University Medical Center Hamburg-Eppendorf, Hamburg, Germany; 2 III. Department of Medicine, University Medical Center Hamburg-Eppendorf, Hamburg, Germany; 3 Department of Psychosomatic Medicine and Psychotherapy, University Medical Center Hamburg-Eppendorf, Hamburg, Germany

**Keywords:** Bariatric surgery, Obesity, Follow-up studies, Health services needs and demand, Treatment outcome

## Abstract

**Introduction:**

Currently, metabolic bariatric surgery (MBS) is the most effective treatment for patients with obesity. While lifelong follow-up care is recommended to support long-term outcomes, follow-up attendance remains suboptimal. With the aim to identify starting points to improve follow-up attendance this study assessed unmet supportive care needs in patients following MBS.

**Materials and Methods:**

Patients who had undergone MBS at a university medical center and attended at least one follow-up appointment were invited to complete an adjusted version of the Supportive Care Needs Survey- Short Form (SCNS-SF34) questionnaire online, along with sociodemographic and medical data.

**Results:**

A total of 327 patients were contacted, of whom 260 participated in the study (participation rate 79.5%). The adapted SCNS-SF34 demonstrated good structural coherence and internal consistency in this bariatric population. Reported needs scores ranged from 17.3 for physical and daily living needs to 26.1 for health system and information needs. In within-person comparisons, health system and information needs were significantly higher than all other domains (all *p*<0.001). Patients with high unmet needs had significantly lower body weight (mean 147kg vs. 156kg, *p*=0.005) at the time of surgery and were more frequently female (77.7% vs. 59.2%, p<0.001).

**Conclusion:**

Health system and information needs represent the most prominent unmet needs after MBS. Routine follow-up care should therefore place greater emphasis on structured patient education, clear communication about available healthcare resources, and individualized information delivery to better address these needs.

## Key Points

The adapted SCNS-SF34 demonstrated good structural coherence and internal consistency in MBS patients.

Detected needs were similar to those reported in other chronic disease populations.

The highest unmet needs were observed in the health system and information domain.

Improving patient education on follow-up care may enhance long-term adherence and outcomes after MBS.

## Introduction

The global prevalence of obesity has increased markedly over recent decades and is associated with substantial morbidity and mortality [1–3]. MBS is among the most effective treatments for obesity and its related comorbidities, including type 2 diabetes, cardiovascular disease, and metabolic dysfunction–associated steatotic liver disease (MASLD) [4–9]. In Germany, MBS is indicated for patients with a body mass index (BMI) ≥ 40 kg/m² or ≥ 35 kg/m² in the presence of obesity-related comorbidities [10]. Postoperative monitoring is recommended by current guidelines to identify potential complications, such as nutritional deficiencies, and optimize long-term outcomes following MBS [10, 11]. An optimal loss to follow-up rate is generally considered to be below 20% [12]. However, follow-up attendance after MBS declines steadily over time, with fewer than 50% of patients typically attending follow-up appointments a few years after MBS [13–17]. Lower follow-up care attendance has been associated with younger age, male sex, avoidant attachment style, and unemployment [18–20]. In contrast, evidence regarding the influence of geographical distance from the treatment center remains inconsistent across studies [21, 22]. Evidence suggests that regular follow-up is associated with improved weight-loss outcomes [11, 23]. To our knowledge, the specific elements of follow-up care that contribute to improved outcomes have not yet been systematically evaluated using standardized patient-reported outcome measures. This highlights the importance of identifying patient-reported needs and barriers to follow-up attendance in order to inform and optimize long-term postoperative care strategies. Previous studies explored patients’ expectations and experiences regarding follow-up care after MBS [22, 24–26]. Despite these insights no standardized, quantitative assessment of supportive care needs exists in the context of MBS follow-up.

The Supportive Care Needs Survey- Short Form (SCNS-SF34) is a validated questionnaire, initially developed to evaluate supportive care needs among cancer patients [27]. Its structure and domains are equally relevant for individuals undergoing complex, long-term treatments such as MBS. We therefore adapted the SCNS-SF34 to the context of MBS while preserving its core validated constructs to systematically evaluate unmet needs across key domains and inform more tailored, patient-centered follow-up strategies.

## Methods

Patients who had undergone MBS at our Center of Excellence for Metabolic Bariatric Surgery were contacted by phone and screened for eligibility based on the following inclusion criteria: age ≥ 18 years, with no upper age limit applied, surgery performed between April 2016 and December 2022, attendance of at least one follow-up visit, fluency in German, internet access, and consent to participate in both our biobank and the present study. The biobank collects biomaterial and associated clinical data from patients undergoing MBS at our center to support translational and clinical research. Participation in the present study required prior consent for inclusion in the biobank. Patients were excluded if they did not meet the above inclusion criteria. Preoperative preparation was individualized based on clinical indication and prior conservative treatment history. Some patients proceeded directly to MBS, whereas others underwent a structured 6-month preoperative program including nutritional counseling and recommended regular independent physical exercise prior to surgery [28]. Additionally, alcohol use disorder was an exclusion criterion for MBS in our center. Therefore, patients with diagnosed alcohol dependence were not eligible for inclusion. Recruitment was conducted by three of the authors (J.W., M.R., and F.B.) between June 2022 and July 2023. Participants received a brief explanation of the study objectives, procedures, and the importance of participation prior to enrollment. Eligible and consenting individuals were sent a link to the online questionnaire (LimeSurvey, Version 5). Data on anthropometric characteristics (height, weight, body mass index (BMI)), comorbidities (type 2 diabetes (T2D), hypertension, obstructive sleep apnea (OSA), dyslipidemia, depression, anxiety disorder) and type of surgery were routinely collected as part of our follow-up care. The institutional review board approved the study and all patients provided informed consent.

### Supportive Care Needs Survey- Short Form (SCNS-SF34)

For this study, the SCNS-SF34 was adapted to the context of MBS and obesity care by replacing references to “cancer” with “obesity” or “metabolic bariatric surgery,” while preserving the original structure and intent of each item (see Supplementary Materials). With a total of 34 items, the SCNS-SF34 covers five domains of supportive care needs: physical and daily living, psychological, sexuality, patient care and support, and health system and information needs. The physical and daily living domain addresses functional limitations, including pain, fatigue, and challenges with daily tasks or household activities. The psychological domain captures emotional and mental well-being, including anxiety, low mood, worries about disease progression, and uncertainty about the future. The sexuality domain encompasses changes in sexual feelings, sexual functioning, or intimate relationships. The patient care and support domain includes needs such as having more choice regarding treatment providers, access to appropriate specialists, and having healthcare staff respond promptly to physical concerns. The health system and information domain reflects needs related to communication and care navigation, such as receiving clear written information about important aspects of treatment and follow-up, guidance on managing symptoms at home, feeling treated as an individual rather than a case, and being kept informed about the status of one’s condition. Each item has to be rated on a 5-point Likert scale (1 = no need/not applicable, 5 = high unmet need). Domain scores were calculated by averaging the responses within each domain and linearly transforming them to a 0–100 scale, with higher scores indicating greater unmet needs. The original English version of the adapted SCNS-SF34 was translated into German by the study team for use in this project. The questionnaire was administered in German using a translated version of the instrument. The questionnaire was self-administered by the patients without assistance from clinicians or researchers. All participants were fluent in German and therefore able to complete the questionnaire independently.

### Statistics

We performed statistical analysis with Stata (StataCorp, version 18 and 19) and GraphPad Prism (GraphPad Software, Inc., version 10). Patient characteristics are presented overall as mean ± standard deviation (SD) for continuous and frequencies/percentages for categorical variables. Psychometric properties of the adapted SCNS-SF34 were examined. The suitability of the data for factor analysis was assessed using Bartlett’s test of sphericity and the Kaiser–Meyer–Olkin (KMO) measure. Structural validity was evaluated by exploratory factor analysis using principal factor extraction with oblique rotation. The number of factors retained was based on eigenvalues, inspection of the scree plot, and the theoretical structure of the original SCNS. Factors were labelled according to item content. Internal consistency of each factor was assessed using Cronbach’s alpha. To compare levels of unmet needs across SCNS-SF34 subscales within individuals, a fixed-effects linear regression model was applied. The model included subscales as categorical predictors and controlled for individual-level heterogeneity by using patient ID as the panel variable. In line with established scoring procedures, participants were categorized to those with and without unmet supportive care needs (1–3 = “low need” vs. ≥4 = “high need”) in any SCNS-SF34 domain. Comparisons between patients with and without unmet needs were conducted using chi-square tests for categorical variables and two-sample *t*-tests for continuous variables. Differences in mean SCNS-SF34 domain scores between postoperative year groups were analyzed using one-way analysis of variance (ANOVA) with Bonferroni correction for multiple comparisons.

## Results

### Patient Characteristics

A total of 327 patients were contacted, of whom 260 completed the questionnaire in full, corresponding to a participation rate of 79.5%. As all included participants completed the questionnaire in full, no item-level missing data were present, and no imputation procedures were required for domain score calculation. The mean age of participants was 44 ± 11.1 years, and the majority were female (68%, *n* = 221). The mean preoperative weight was 151 ± 27.1 kg, corresponding to a mean BMI of 50.5 ± 7.9 kg/m². Surgical procedures included laparoscopic sleeve gastrectomy, Roux-en-Y gastric bypass (RYGB), and other bariatric techniques. At the time of data collection, participants had attended an average of 3.3 follow-up visits (Table 1), corresponding to a follow-up rate of 72.5%. The duration of postoperative follow-up ranged from 3 months to 6 years, with most patients in their first (25.7%), second (32.7%), or third (32.4%) postoperative year. Except for a significantly higher proportion of patients with hypertension among responders, baseline characteristics were largely comparable between responders and non-responders (Table 2). Follow-up attendance was significantly lower among male patients compared to female patients (67.5% vs. 74.9%, *p* = 0.01).

**Table 1:** Preoperative characteristics and average follow-up attendance of all contacted patients

Characteristic	
Contacted patients[n]	327
Age [years]	44 ± 11.1
Weight [kg]	151 ± 27.1
Height [cm]	173 ± 9.5
BMI [kg/m^2^]	50.5 ± 7.9
Sex [n female/%]	221/67.6
Average attended follow-up appointments [n]	3.3 ± 1.3
MBS procedure [n/%] • Sleeve gastrectomy • RYGB • Others	256/78.3 65/19.9 6/1.8
Type 2 diabetes [n/%]	100/30.6
Hypertension [n/%]	177/54.1
OSA [n/%]	71/21.7
Dyslipidemia [n/%]	166/50.8
Depression [n/%]	52/15.9
Anxiety disorder [n/%]	11/3.4
Smoking status [n/%] ^1^ • Current smoker • Former smoker • Non-smoker	76/24.5 29/9.4 205/66.1
Type of preoperative preparation [n/%] ² • Direct surgery • Structured conservative program • External preoperative preparation	195/63.3 100/32.5 13/4.2

^1^ Percentages are calculated excluding missing data. Missing values: *n* = 17

² Percentages are calculated excluding missing data. Missing values: *n* = 19

**Table 2:** Preoperative characteristics of responders and non-responders and average follow-up appointments

Characteristic	Responders	Non-responders	*p*-value
Patients[n]	260	67	
Age [years]	44.6 ± 10.9	42.2 ± 11.8	0.11
Weight [kg]	151 ± 27	155 ± 27.5	0.33
Height [cm]	173 ± 9.3	173 ± 10.3	0.99
BMI [kg/m^2^]	50.3 ± 7.9	51.3 ± 7.5	0.35
Sex [n female/%]	178/68.4	43/64.2	0.5
Average attended follow-up appointments [n]	3.3 ± 1.3	3.1 ± 1.5	0.32
MBS procedure [n/%] • Sleeve gastrectomy • RYGB • Others	201/77.3 54/20.8 5/1.9	55/82.1 11/16.4 1/1.5	0.12
Type 2 diabetes [n/%]	83/31.9	17/25.3	0.3
Hypertension [n/%]	151/58.1	26/38.8	0.005
OSA [n/%]	59/22.7	12/17.9	0.4
Dyslipidemia [n/%]	134/51.5	32/47.8	0.58
Depression [n/%]	37/14.2	15/22.4	0.1
Anxiety disorder [n/%]	8/3.1	3/4.5	0.57
Smoking status [n/%] ^1^ • Current smoker • Former smoker • Non-smoker	56/22.7 23/9.3 168/68	20/31.8 6/9.5 37/58.7	0.31
Type of preoperative preparation [n/%] ² • Direct surgery • Structured conservative program • External preoperative preparation	152/62 82/33.5 11/4.5	43/68.2 18/28.6 2/3.2	0.644

^1^ Percentages are calculated excluding missing data. Missing values: *n* = 17

² Percentages are calculated excluding missing data. Missing values: *n* = 19

### Psychometric Evaluation of the Adapted SCNS-SF34

The psychometric properties of the adapted SCNS-SF34 were evaluated prior to outcome analyses. The data were suitable for factor analysis, as indicated by a significant Bartlett’s test of sphericity (*p* < 0.001) and an excellent sampling adequacy with a Kaiser–Meyer–Olkin (KMO) of 0.924. Exploratory factor analysis using principal factor extraction with oblique rotation supported a five-factor solution. The retained factors accounted for 91.7% of the common variance. Based on item content, the five factors were interpreted as health system and information needs, psychological needs, sexuality needs, patient care and support needs, and physical and daily living needs. Overall, factor loadings were high and indicated a clear clustering of items within domains. While several factors closely resembled the original SCNS-SF34 structure, some overlap between patient care and support needs and health system and information needs was observed, suggesting partially altered item–domain relationships in the MBS population. Detailed rotated factor loadings and uniqueness values are reported in Supplementary Table S1. Internal consistency was good to excellent across factors (Cronbach’s alpha range: 0.838–0.944). Details of the factor structure and reliability estimates are presented in Table 3.

**Table 3:** Internal consistency and exploratory factor analysis of the adapted SCNS-SF34

Factor	Domain	Number of items	Items	Cronbach’s α
1	Health system and information needs	11	24–34	0.9295
2	Psychological needs	10	6–15	0.9154
3	Sexuality needs	3	16–18	0.9441
4	Patient care and support needs	5	18–23	0.8991
5	Physical and daily living needs	5	1–5	0.8383

### Patients’ Needs by Subcategory

**Fig. 1:**
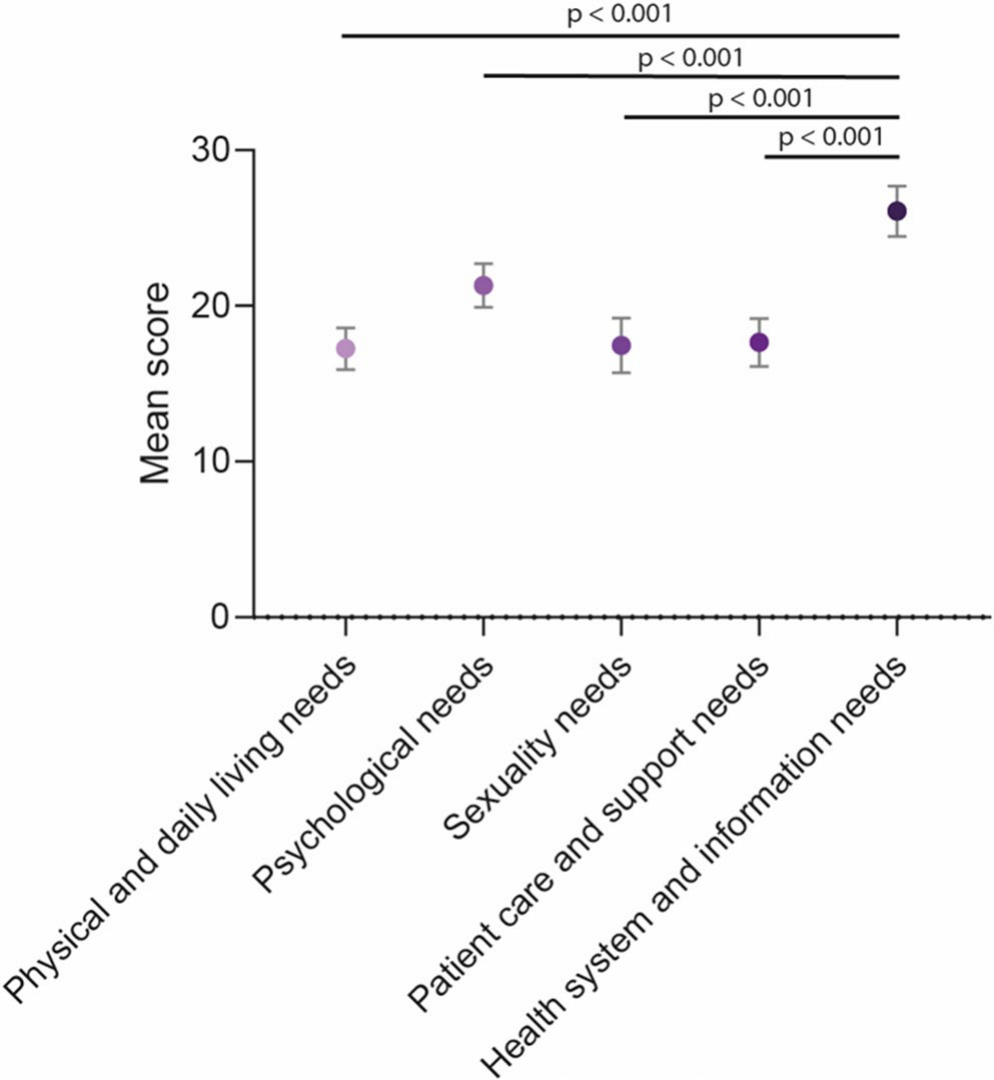
Mean scores for each SCNS-SF34 domain. Data are presented as mean ± standard deviation. Statistical analysis was performed using a fixed-effects linear regression model to compare domain scores within individuals. Compared with all other domains, health system and information needs showed significantly higher scores (all *p* < 0.001)

The mean score for physical and daily living needs was 17.3 ± 21.8, for psychological needs 21.3 ± 22.5, for sexuality needs 17.5 ± 28.1, for patient care and support needs 21.3 ± 22.5, and for health system and information needs 26.1 ± 26.0. A fixed-effects linear regression model was then applied to compare need levels within individuals. Health system and information needs were significantly higher as compared to all other domains (Fig. 1).

### Patients’ Needs by Subcategory and Postoperative Year

**Fig. 2:**
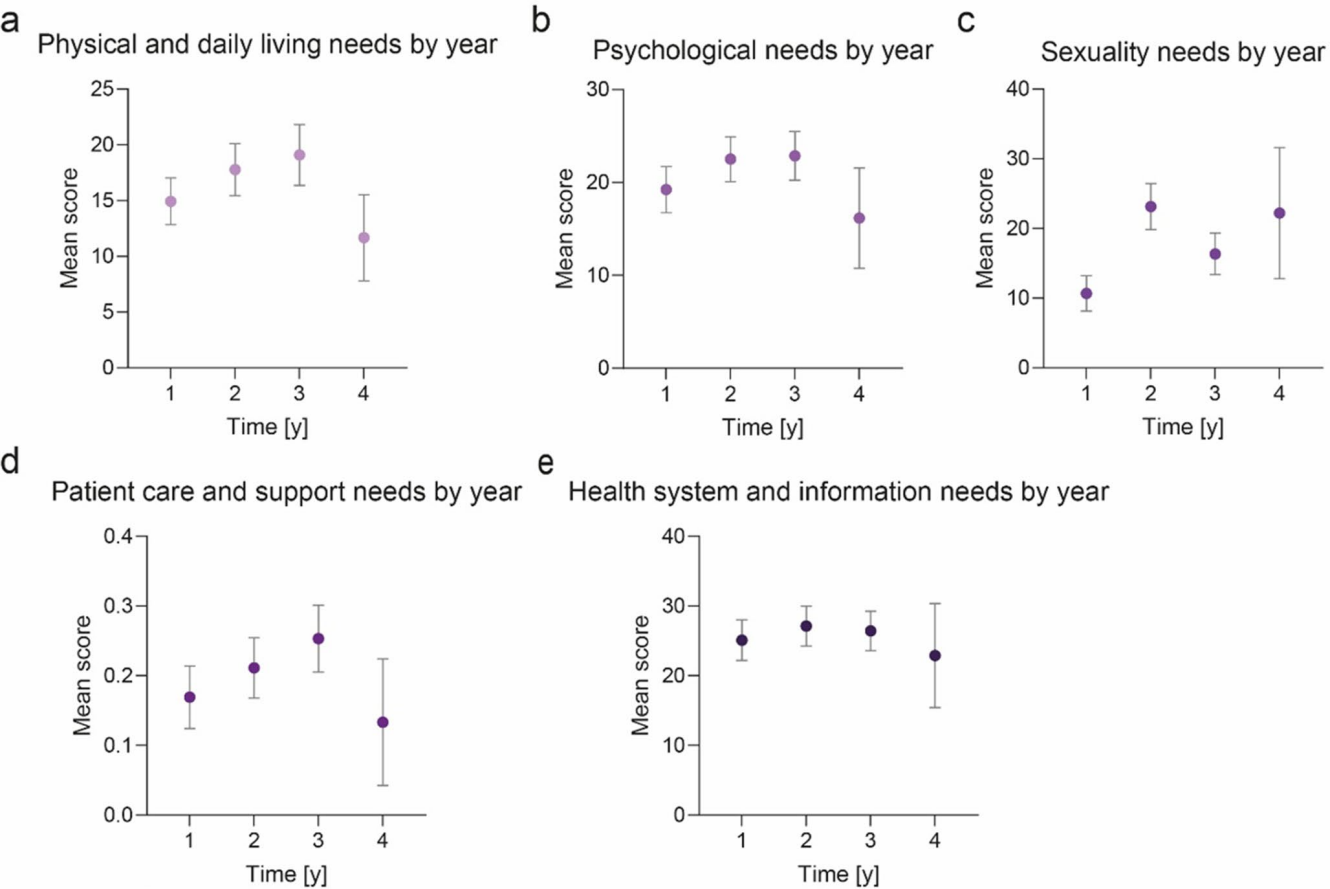
Mean SCNS-SF34 domain scores for physical and daily living needs (**a**), psychological needs (**b**), sexuality needs (**c**), patient care and support needs (**d**) and health system and information needs (**e**) by postoperative year. Data are presented as mean ± standard deviation. Statistical analysis was performed using one-way analysis of variance (ANOVA) with Bonferroni correction for multiple comparisons

When comparing patients across postoperative years, physical and daily living needs were increasing in the first three years and lower in later years; however, these differences were not statistically significant. (Fig. 2a). A similar pattern was observed for psychological needs, with no significant differences across years (Fig. 2b). Sexuality-related needs showed a temporary increase in the second postoperative year before decreasing again, but this variation was not significant (Fig. 2c). Patient care and support needs followed a trend comparable to physical and daily living needs, rising up to three years postoperatively without reaching statistical significance (Fig. 2d). Lastly, health system and information needs remained consistently high throughout the observation period (Fig. 2e).

### Comparison of Patients‘ Characteristics with and Without Unmet Needs

Compared with patients without high needs, those with unmet needs had a significantly lower preoperative weight (147 ± 25.5 kg vs. 156 ± 27.7 kg, *p* = 0.005) and were significantly shorter (172 ± 8.7 cm vs. 174 ± 9.9 cm, *p* = 0.018), although no significant difference in BMI was observed (*p* = 0.066). In addition, patients with high needs were more frequently female (77.7% vs. 59.2%, *p* < 0.001) and had a higher prevalence of depression (18.2% vs. 8.9%, *p* = 0.033) (Table 4). The distribution of preoperative preparation type did not differ significantly between patients with low and high unmet needs (*p* = 0.239). No significant differences in follow-up visit frequency were observed between low- and high-need groups, either overall or after stratification by sex (all *p* > 0.05). The proportion of patients with high unmet needs did not differ significantly between sleeve gastrectomy and gastric bypass procedures (Table 4). Likewise, no significant differences in domain scores were observed between procedures (all *p* > 0.05).

**Table 4:** Comparison of patient’s characteristics with and without unmet needs

Characteristic	High needs	Low needs	*p* value
Patients[n]	148	112	
Age [years]	44.6 ± 10.9	44.6 ± 11	0.954
Weight [kg]	147 ± 25.5	156 ± 28	0.007
Height [cm]	172 ± 8.8	175 ± 9.7	0.006
BMI [kg/m^2^]	49.6 ± 7.2	51.1 ± 8.6	0.138
Sex [n female/%]	115/77.7	63/56.3	< 0.001
Average attended follow-up appointments [n]	3.3 ± 1.2	3.3 ± 1.3	0.911
MBS procedure [n/%] • Sleeve gastrectomy • RYGB • Others	108/73 37/25 3/2	93/83 17/15.2 2/1.8	0.148
Type 2 diabetes [n/%]	50/33.8	33/29.5	0.72
Hypertension [n/%]	86/58.1	65/58	0.991
OSA [n/%]	32/21.6	27/24.1	0.636
Dyslipidemia [n/%]	78/52.7	56/50	0.666
Depression [n/%]	27/18.2	10/8.9	0.033
Anxiety disorder [n/%]	7/4.7	1/0.9	0.076
Smoking status [n/%] ^1^ • Current smoker • Former smoker • Non-smoker	33/23.2 11/7.8 98/69	23/21.9 12/11.4 70/66.7	0.614
Type of preoperative preparation [n/%] ² • Direct surgery • Structured conservative program • External preoperative preparation	85/60.7 46/32.9 9/6.4	67/63.8 36/34.3 2/1.9	0.239

^1^ Percentages are calculated excluding missing data. Missing values: *n* = 13

² Percentages are calculated excluding missing data. Missing values: *n* = 15

## Discussion

In this study, we systematically assessed supportive care needs of patients after MBS using a modified version of the SCNS-SF34. As the instrument was originally developed and validated in oncology populations, no prior formal validation existed for use in MBS patients. Our internal psychometric evaluation demonstrated a coherent five-factor structure and good internal consistency across domains. Although the overall structure was largely comparable to the original instrument, some overlap between domains suggests that supportive care needs in an MBS population may not entirely mirror those observed in cancer patients. While these findings indicate acceptable structural coherence and internal reliability in our cohort, they do not replace a comprehensive validation process. Future studies should therefore aim to perform confirmatory factor analyses and external validation in independent MBS populations.

The highest needs were detected in the health system and information domain, indicating that informational and system-related aspects of care remain key areas for improvement. Overall, the pattern and relative magnitude of supportive care needs observed in our cohort appear similar to those reported in studies of cancer patients, although direct comparability is limited due to the lack of prior formal validation of the adapted instrument in MBS populations [29, 30]. Consequently, our findings primarily allow for internal comparisons within the present cohort rather than definitive cross-population comparisons. While the observed structural coherence and internal consistency suggest that the adapted version functions adequately in this context, a comprehensive external validation study is required to confirm its validity in MBS patients. Taking these considerations into account, health system and information needs emerged as the most prominent domain in our cohort, whereas previous qualitative studies have highlighted psychological needs as particularly pronounced [31–34]. This discrepancy might be due to the quantitative assessment of needs in the present study and qualitative study designs in previous investigations. Given that health system and information needs emerged as the most prominent domain in our cohort, the role of structured preoperative counseling warrants consideration. Current perioperative care recommendations, including enhanced recovery protocols, emphasize patient information and education as integral components of bariatric surgical care [35]. Moreover, a contemporary survey of MBS programs highlights the perceived importance of structured patient education in optimizing postoperative engagement and outcomes [36]. However, in our cohort, we did not observe a significant association between the type of preoperative preparation and postoperative supportive care needs. This may reflect heterogeneity in counseling content, variability in patient engagement, or the possibility that postoperative informational needs evolve independently of preoperative education. Prospective studies are needed to better delineate the longitudinal impact of structured preoperative counseling on postoperative supportive care needs.

Our second major finding was that supportive care needs remained largely stable across postoperative years, suggesting that unmet needs persist throughout the course of follow-up after MBS. This stability of needs over time is consistent with existing literature and with our previous qualitative work, suggesting that although patients generally have realistic expectations regarding follow-up care, many do not fully recognize the importance of consistent long-term follow-up and that patient education could aid improving adherence rates [24]. Similar patterns have also been reported in patients with other chronic diseases. For example, a study from Finland demonstrated that improved patient education delivered in general practice settings was associated with better medication adherence [37]. Additionally, two earlier randomized controlled trials demonstrated that patient education improved clinical outcomes in both hypertension and schizophrenia [38, 39]. Therefore, it may be beneficial to strengthen patient education on the importance of consistent follow-up both before and after surgery. Further research is warranted, and prospective studies are needed to confirm the impact of enhanced education on long-term follow-up adherence.

Interestingly, patients with high levels of unmet needs were more frequently female. This finding is consistent with recent literature showing that female patients in oncology settings also report higher levels of supportive care needs [40–42]. This is important to consider in clinical practice, as female patients may require more support during follow-up care.

This study has several strengths and limitations. Strengths include the relatively large sample size and the systematic assessment of patient-reported outcomes in a real-world clinical setting. Limitations include the cross-sectional design and the fact that the study was conducted at a single center, which may affect generalizability. Although our analyses support the structural validity and internal consistency of the adapted SCNS-SF34, the instrument has not undergone a comprehensive validation process for metabolic bariatric populations. In particular, confirmatory factor analysis, test–retest reliability, and external validation in independent cohorts were not performed. Furthermore, supportive care needs specific to obesity and MBS may not be fully captured by an instrument originally developed for oncology settings. Therefore, the findings should be interpreted with appropriate caution, and further psychometric evaluation in dedicated metabolic bariatric samples is warranted. A further limitation relates to the inclusion criterion requiring attendance at least one postoperative follow-up visit. By design, patients who were completely lost to follow-up were not included in the analysis. This may introduce selection bias, as individuals disengaged from follow-up care could represent a subgroup with greater barriers to access, lower adherence, or higher levels of unmet supportive care needs. Consequently, the supportive care needs reported in this study may underestimate the true burden in the overall MBS population. Future research should specifically investigate patients lost to follow-up to better understand structural, psychosocial, and healthcare-related barriers. Such data would be essential for developing targeted outreach strategies and improving long-term postoperative care pathways.

## Conclusion

In conclusion, this study provides the first quantitative assessment of supportive care needs in patients after MBS. Health system and information needs emerged as the most prominent unmet areas, underscoring the importance of clear communication, structured postoperative education, and coordinated long-term support. To address these informational deficits, standardized follow-up protocols incorporating scheduled, content-focused counseling sessions and accessible written or digital information resources may help improve patient understanding and engagement. Future research should focus on validating the adapted SCNS-SF34 in metabolic bariatric populations and on developing targeted interventions to improve patient-centered follow-up care.

## Data Availability

The datasets used and/or analysed during the current study are available from the corresponding author on reasonable request.
